# Comparison of lithotripsy methods during mini-PNL: is there a role for ballistic lithotripsy in the era of high-power lasers

**DOI:** 10.1186/s12894-024-01443-6

**Published:** 2024-03-07

**Authors:** Muhammed Arif Ibis, Ahmet Furkan Özsoy, Mehmet Fatih Özkaya, Emre Erdem, Serhat Erkmen, Ahmet Doruk Güler, Mehmet İlker Gökce

**Affiliations:** Department of Urology, Ankara University School of Medicine, Adnan Saygun Caddesi, Ankara, Altındağ Turkey

**Keywords:** Ballistic lithotripsy, Kidney stone, Laser lithotripsy, mini-PNL, Percutaneous nephrolithotomy

## Abstract

**Background:**

For renal stones > 20 mm, percutaneous nephrolithotomy (PNL) offers the best stone clearance rates with acceptable complication rates. This study aimed to compare the efficiency of high-power holmium YAG laser and ballistic lithotripsy during mini-PNL.

**Methods:**

Data from 880 patients who underwent mini-PNL for renal stones was investigated retrospectively. The study utilized propensity score matching to create two groups: laser lithotripsy (*n* = 440) and ballistic lithotripsy (*n* = 440). The groups were matched based on stone size, Guy’s stone score, and stone density. The main objectives of the study were to assess the stone-free rate (SFR), duration of surgery, and complication rates.

**Results:**

The average age of the population was 51.4 ± 7.1 years, with a mean stone size of 28.6 ± 8.3 mm and a mean stone density of 1205 ± 159 HU. There were no significant differences between the groups. The SFRs of the laser lithotripsy and ballistic lithotripsy were 92.5% and 90.2%, respectively (*p* = 0.23). The laser lithotripsy group had a notably shorter surgery time (40.1 ± 6.3 min) compared to the ballistic lithotripsy group (55.6 ± 9.9 min) (*p* = 0.03). Complication rates were similar (*p* = 0.67).

**Conclusions:**

Our study shows that a high-power holmium YAG laser provides quicker operation time compared to ballistic lithotripsy. However, ballistic lithotripsy is still an effective and safe option for stone fragmentation during mini-PNL. In places where a high-power holmium YAG laser is not available, ballistic lithotripters are still a safe, effective, and affordable option for mini-PNL.

## Background

For renal stones > 20 mm, percutaneous nephrolithotomy (PNL) offers the best stone clearance rates with acceptable complication rates [1–3]. Miniaturization of the percutaneous tract during PNL reduces the risk of complications and Jackman et al. reported the first mini-PNL series [4]. By the development of mini-PNL systems the outcomes of the mini-PNL procedure got better [5–9] and it is applied more commonly [10]. The latest technological developments in mini-PCNL techniques are promising; however, further research is needed to ensure safety and efficacy. Uncovering more efficient techniques in a shorter period through studies can provide urologists with a personalized approach to stone management [11–13].

Following percutaneous access, the stones in the collecting system need to be fragmented [14]. Since the inception of the PNL, various intracorporeal lithotripters have been developed for the fragmentation of kidney stones. These lithotripters vary in terms of energy sources, including electrohydraulic, pneumatic, ultrasonic, and laser lithotripsy, depending on the type of energy utilized. Currently, Holmium: Yttrium-Aluminum-Garnet (Ho:YAG) laser is widely accepted as the gold standard laser lithotripsy for mini-PNL [15].

The introduction of high-power holmium YAG lasers into the market with technological advancements has facilitated their utilization in surgeries for prostate and kidney stones. In generally, holmium laser systems with a power of 100 watts and above are classified as high-power systems [16]. At the present, a high-power holmium YAG laser provides effective fragmentation of the stones during the surgery. In a previous study, we compared the efficacy of the low-power holmium YAG laser with a ballistic lithotripter during a mini-PNL procedure. In this study, a combination of ballistic and low-power holmium YAG laser was found to decrease the operation times when compared to the use of both technologies alone [17]. Ganesamoni et al. also prospectively compared laser and ballistic lithotripters and concluded that both methods are safe and effective. However, in this study, a low-power holmium YAG laser was used [18].

However, the current existing literature lacks studies comparing the efficacy and safety of high-power holmium YAG lasers with ballistic lithotripters during mini-PNL and this study aimed to compare the efficiency of high-power holmium YAG laser and ballistic lithotripsy.

## Methods

In this study, the data of 2563 patients who underwent mini-PNL at our institution between January 2014 and December 2023 was investigated retrospectively. We identified 440 patients who underwent ballistic lithotripsy and 793 patients who underwent high-power holmium YAG lithotripsy during mini-PNL. The laser lithotripsy (*n* = 440) and ballistic lithotripsy (*n* = 440) groups were formed using the propensity score matching technique. Matching criteria included stone size, stone density, and Guy’s stone score (GSS). Cases involving anatomic abnormalities and pediatric cases were excluded. Approval for the study was obtained from our institution’s ethical committee, with a registration number of 05-299-18.

The analyzed parameters included age, gender, stone size, stone density (measured in Hounsfield Units - HU), GSS, duration of operation and hospitalization, complication rates, and stone-free rates (SFR). For cases with multiple stones, the sum of the largest diameters of all stones was calculated. Postoperative imaging involved KUB and/or ultrasonography, and a non-contrast computer tomography (CT) scan was conducted if there was suspicion of any residual fragments before JJ stent extraction between postoperative days 7–15. SFR was defined as the absence of any size of residual fragments. Post-operative complications were assessed using the Clavien–Dindo grading system.

### Surgical method

Patients were positioned in the Galdakao modified supine Valdivia (GMSV) position [19], and a 6Fr ureteral catheter was inserted for a retrograde pyelogram. Percutaneous access was established with the guidance of fluoroscopy and ultrasound. The MIP-M kit (Karl Storz, Tuttlingen, Germany) was used to create a percutaneous tract with 15 Fr metallic dilation and a 16 Fr metallic sheath was placed. A 12 Fr nephroscope was inserted, and laser lithotripsy was conducted using Dornier Medilas H140 (Wessling, Germany) or Potent HZ 90–120 (Guangzhou, China) with fragmentation settings of 1.5–2.5 J and 10–30 Hz. Ballistic lithotripsy (Lithobox or Lithobox Zero İnceler Medikal, Ankara, Turkey) was performed using a 4 Fr probe with a frequency of 6–10 Hz. The fragments were removed using the vacuum cleaner effect, and a basket was employed when necessary. The decision to place a JJ stent was made as an exit strategy by the operating surgeon. A nephrostomy catheter was not routinely inserted in any patient.

### Statistical analysis

Continuous data were conveyed as mean with accompanying standard deviation and categorical data were represented by numbers and their standard deviations. Normal distribution assumption for continuous variables was analyzed with the Shapiro-Wilk test. Student’s t-test and Mann-Whitney U test were utilized for comparing continuous variables, while the Chi-square test was applied to assess differences in categorical variables across the two groups. A value of *p* < 0.05 was considered statistically significant.

## Results

The average age of the study population was 51.4 ± 7.1 years, with a mean stone size of 28.6 ± 8.3 mm and a mean stone density of 1205 ± 159 HU. 47% of the patients, namely 420 individuals, consisted of females out of a total of 880 patients. The groups exhibited similarity in terms of age, gender, stone size, stone density, and Guy’s stone score. The findings are outlined in Table 1.

**Table 1:** Demographic and patient-related characteristics of the groups

Parameter	Laser lithotripsy (*n* = 440)	Ballistic lithotripsy (*n* = 440)	p value
Age (mean ± SD)	51.5 ± 8.2	51.3±8.6	0.77
Gender, n (%)			0.78
Male	228 (51.8)	232 (52.7)	
Female	212 (48.2)	208 (47.3)	
Stone size, mm (mean ± SD)	28.6±8.5	28.6±8.3	1
Stone density, HU, (mean ± SD)	1205±103	1205±110	1
Guys stone score (GSS), n (%)			1
GSS-1	287 (65.3)	287 (65.3)	
GSS-2	114 (25.9)	114 (25.9)	
GSS-3	39 (8.8)	39 (8.8)	
GSS-4	-	-	

*SD*: standard deviation; *HU*: Hounsfield unit

The SFRs for laser lithotripsy and ballistic lithotripsy were 92.5% and 90.2%, respectively, with no statistically significant difference (*p* = 0.23). The duration of surgery was notably shorter in the laser lithotripsy group (40.1 ± 6.3 min) compared to the ballistic lithotripsy group (55.6 ± 9.9 min), with a significance level of *p* = 0.03. Both groups had a median postoperative hospital stay of one day (*p* = 0.99). Similar complication rates were observed in the two groups (*p* = 0.67), with all complications observed as Calvien-Dindo Grade I and II. Transfusion was necessitated for two patients in the laser lithotripsy cohort and a single patient in the ballistic lithotripsy group. Grade I complications involved postoperative antipyretic or analgesic therapy (Table 2).

**Table 2:** Comparison of groups for surgical outcomes

Parameter	Laser lithotripsy (*n* = 440)	Ballistic lithotripsy (*n* = 440)	p value
Stone free rate, n (%)	407 (92.5)	397 (90.2)	0.23
Complication rate, n (%)	25 (11.5)	22 (8.6)	0.65
Grade I	23	20	
Grade II	2	1	
Duration of surgery, minutes (mean ± SD)	40.1±6.3	55.6±9.9	0.03
Hospital stay, days (median &range)	1 (1–3)	1 (1–3)	0.99

*SD*: standard deviation

## Discussion

Mini-PNL is a safe and effective treatment modality for the management of renal stones. It provides excellent stone-free rates with less complication rates compared to standard PNL [10, 20]. The effectiveness of the mini-PNL procedure relies on efficient lithotripsy as the nature of the procedure relies on fine fragmentation of the stones compared to standard PNL. Holmium laser is the most widely applied lithotripter in mini-PNL with its high peak power and fragmentation capacity. However, ballistic lithotripters are still a good choice especially when the stone burden is huge and and when the stone density is high. However, we found out that a high-power holmium YAG laser maintains shorter operative times with similar success and complication rates compared to the ballistic lithotripters.

Holmium YAG laser lithotripsy can be applied with thin fibers that can work through the narrow working channels of the miniaturized nephroscopes. This provides the advantage of good irrigation during the procedure. Ultrasonic lithotripters that also provide suction to the tiny fragments require huge probes up to 3.3 mm in diameter to work efficiently. The small diameter ultrasonic lithotripsy probes such as 1.5 mm can fragment the stone but the suction capacity is limited. Therefore, ballistic probes still have a role during mini-PNL with the available 1.3 mm probes. Another advantage of holmium YAG lithotripsy is the possibility of adjusting the laser parameters concerning the characteristics of the stones.

Ballistic lithotripsy needs direct contact of the probe with the stone for fragmentation and the main disadvantage of this method is stone retropulsion, especially in the case of dilated collecting systems [21, 22]. Also in the case of an impacted stone, with the mechanical effect of the ballistic probe, the mucosa can get damaged and this can result in mucosal bleeding or migration of the stone out of the collecting system. During mini-PNL fragment extraction relies on Bernoulli’s principle so-called Vacuum-Cleaner Effect [23]. Laser lithotripsy is the best method to apply the Vacuum-Cleaner Effect as the surgeon does not need to take out the laser fiber out of the working channel of the nephroscope.

In a recent study, the use of high-power holmium laser during ureteroscopy has demonstrated a success rate of 84.5% in patients with large, bilateral, or multiple stones [24]. Additionally, compared to low-power lasers, high-power lasers have been noted to reduce operation time and achieve higher success rates in retrograde intrarenal surgery for pediatric patients [25]. Recently, the ultra-mini PNL method, utilizing a 120-watt Holmium: YAG laser, has been reported with a success rate of 91.6% [26]. Another study showed complete stone clearance in all patients one month after the procedure [27].

Ganesamoni et al. conducted a prospective randomized study involving 60 patients to compare laser and ballistic lithotripsy in mini-PNL. The research revealed that the groups exhibited similarity in total operative time, and no significant differences were observed between them concerning stone fragmentation time, success rates, and complication rates. However, the stone migration rate was higher, fragment retrieval was more difficult in the ballistic lithotripsy group [18]. In the current study, we found out that holmium YAG lithotripsy was faster in terms of operation time compared to ballistic lithotripsy. This can be explained by the use of a high-power holmium YAG laser in our group that provided higher energy for better fragmentation and higher frequency for faster lithotripsy. In the study by Ganesamoni et al. [18] the holmium laser was a 30 Watts device another important difference is the stone diameter in our cohort was greater than the in the study by Ganesamoni et al. (28.6 mm vs. 17.5 mm) and this difference also provided an advantage for the high power holmium lithotripsy group.

In another previous study, Tangal et al. compared the low-power holmium YAG laser with ballistic lithotripsy, and in this study, there was a third group that included patients with combined ballistic and laser lithotripsy [17]. In this study, the combination of the ballistic and holmium YAG laser was more efficient than the application of each lithotripter alone. Also, all the stones were high-density stones and the additional use of ballistic lithotripsy improved the efficiency of low power holmium YAG laser. In this current study, the high-power settings with the holmium YAG laser provided a much more efficient lithotripsy and took out the possible advantage of the ballistic lithotripter.

Unless complications arise, postoperative hospital stays typically range between 1 and 4 days according to current literature [28]. In our study, the duration of hospitalization in both groups was in line with the existing literature. This suggests that it does not contribute to an increase in the loss of workforce in daily life.

An important advantage of ballistic lithotripters is the affordable cost in terms of both the first assembly price and the maintenance costs. A high-power holmium YAG laser is an expensive device and also the costs of the laser fibers are more expensive than a simple ballistic probe. Additionally, the high-power holmium YAG lasers require a special electric supply and cause significant noise in the operating room. Therefore, high-power holmium YAG lasers have specific disadvantages and despite being more efficient than ballistic lithotripters, this older technology still has a role in clinical practice.

Our study has notable limitations, primarily the absence of randomization and the retrospective nature of data analysis. Furthermore, the individual time for fragmentation was not recorded; instead, we have data on total operative times. Despite these limitations, the groups were carefully matched based on stone size and stone density. Thus, we contend that any potential selection bias affecting fragmentation times is minimized.

## Conclusion

Our study demonstrates that a high-power holmium YAG laser provides quicker operation time compared to ballistic lithotripsy. However, ballistic lithotripsy is still an effective and safe option for stone fragmentation during mini-PNL. In places where a high-power holmium YAG laser is not available, ballistic lithotripters are still a safe and effective option for mini-PNL. However, further advancement in our understanding of this subject necessitates the conduct of randomized controlled prospective studies.

## Data Availability

All datasets used and analyzed during the current study are available from the corresponding author upon reasonable request.

## Abbreviations

CT: computer tomography
GMSV: Galdakao modified supine Valdivia
GSS: Guy’s stone score
Ho: YAG:Holmium:Yttrium-Aluminum-Garnet
PNL: percutaneous nephrolithotomy
SFR: stone-free rate
